# On Medical Consultations

**Published:** 1808-03

**Authors:** 


### *To the Editors of the Medical and Physical Journal.*

GENTLEMEN,

AMONG the almost innumerable professors of the science of medicine, a considerable disparity of judgment and professional skill must necessarily exist. Nature has by no means formed us with equal capabilities of improvement, nor are our own exertions at all more proportionate than the endowments of nature. One man is naturally dull; another is habitually indolent; while a third combining the rare requisites of diligence and talent, will probably arrive at much greater excellence than either. Perfection, however, in the present state of medical knowledge, is what no physician, however great his merit, can possibly attain: for after industry and ability have done all that they can, much will still remain undone: doubts and difficulties; misconceptions in theory, and mistakes in practice, will still continue; and new and anomalous appearances will be perpetually occurring to confound the learned and perplex the skilful. A conscientious practitioner, knowing these circumstances, will, in every case of importance, proceed with the utmost diffidence and caution. He will act like a man travelling in a road with which he is but imperfectly acquainted, and which he is not certain will conduct him to the place he would reach. He will look to the right hand, and to the left, to see if any other path presents itself. He will keep the point he would arrive at steadily in view; and so long as the track he follows, appears to lead towards it, he will continue to advance: but upon the first deviation or division, he will pause; take his observations anew; and if an opportunity offers, will immediately apply to some person for direction.

This leads me to the subject of medical consultation: a practice, which, though pretty frequent in the metropolis, is, I apprehend, too much neglected in other parts of the country: though, surely, if it be deemed advisable in the very very focus of medical abilities, it must be doubly so where the limited opportunities of improvement may be supposed to render them somewhat less brilliant. But here it may very properly be asked, in what particular instances a consultation is to be considered necessary; for since diseases continually prove fatal under the direction of the most skilful of the profession, the death of the patient cannot alone afford good reason for questioning the conduct of the physician. To this I answer; that in every anomalous and difficult case, when the danger becomes urgent, and the practitioner finds his judgment wavering and unconfirmed; still more so, when he is conscious of not understanding the disorder at all, he is bound, by every consideration of honour and probity, to advise the calling in of further assistance. And should a young and healthy person, to the astonishment of every body, be unhappily cut off by an *unknown* and *indescribable* disease, without such advice having been given,—the conduct of the medical attendant may, in my opinion, be very fairly suspected; particularly if his abilities be held at all equivocal in the opinion of his professional brethren.

The friends of the sick, who can be no judges either of the talents of the physician, or the danger of the disease, will seldom think a consultation necessary; and motives of delicacy may prevent their calling one when they do. This duty, therefore, rests entirely with the physician himself. It is his business to endeavour by *every possible means* to save the live of the patient, and if he knowingly neglects *any* of these means, he, in my opinion, is morally responsible for the event.

I am aware but of two motives which can render a medical attendant averse to assistance, whenever he finds his own judgment at a stand. The one is, a consciousness of incapacity, which shrinks from the eye of superior intelligence; the other, a fear of advancing the interests of a rival. But what would that man deserve, who, neglecting the first duties of his station, could thus trifle with the lives and happiness of his fellow creatures!—The bare conception of such a character is painful in the extreme. The reality, I hope, has no where an existence.

Incalculable are the advantages which would accrue to society, and the medical profession, from the latter consulting together in all cases of difficulty and danger. It would ensure to the sick the fairest chance of recovery, not only by calling around them a collective force of abilities, but also by rendering the physician first employed extremely tremely cautious and attentive, knowing that his conduct might afterwards be inspected by those who would be able to judge of its propriety. It would open a fine field for improvement, by establishing a kind of clinical ward in private chambers, where each practitioner might profit as well by his own observations, as the judgment and experience of the rest. It would, moreover, afford merit, too often neglected from being unknown, an opportunity of distinguishing itself, and of rising into eminence. But in order to the general adoption of such a plan, it is necessary that it be attended with no additional expence to the sick or their relatives: and therefore I think that every physician or surgeon, (for I would include both branches of the profession,) when called to a consultation, should pay, at least, his first visit, without fee or reward; considering it merely as an act of courtesy to a professional brother.

These remarks, Gentlemen, which I flatter myself you will approve, are not the product of fanciful speculation; but were suggested by some unhappy circumstances which have recently occurred, and through the medium of your Journal may possibly meet the eye of those for whom they are intended.

"Equidem de te dies noctesque, ut debeo, cogitans, casus duntaxat humanos et incertos eventus valetudinis, et naturæ communis fragilitatem extimesco."

*February* 12, 1808.

